# Book Notices

**Published:** 1866-12

**Authors:** 


### EDITORIAL.

### BOOK NOTICES.

*Practical Therapeutics, considered chiefly with reference to articles of the Materia Medica.* By EDWARD JOHN WARING, F. R. C. S., Surgeon in Her Majesty's Indian Army. Philadelphia: Lindsay & Blakiston. 1866.

We have greatly enjoyed the examination of this volume. It is indeed one of the most practical works that has ever attracted our attention. Combining the merits of Wood, Beck, Stille, and the U. S. Dispensatory, it forms a volume which no young physician can afford to be without.

The different articles of the materia medica are considered in alphabetic order, and the volume is concluded by a hundred pages devoted to certain medical agents and classes of medicines which cannot be arranged in any mere list of drugs. A very full index of diseases with their appropriate remedies is appended to the book. The whole book, by the judicious use of small type, is compressed within eight hundred pages, forming the best example of what is intended by the phrase *multum in parvo.* We hope that every medical student will provide himself with a copy of this invaluable treatise before he receives his degree of *Doctor Medicinæ.*

*A Handy Book of Ophthalmic Surgery for the use of Practitioners.* By JOHN Z. LAURENCE F. R. C. S., M. B., Surgeon to the Ophthalmic Hospital, Southwark, etc., etc., and ROBERT C. MOON, House Surgeon to the Ophthalmic Hospital, Southwark. 190 pages. Philadelphia: Henry C. Lea.

For those about entering upon the study of ophthalmology, this little work of Mr. Laurence and Mr. Moon will prove a most valuble text book. Such a work, carefully studied, will furnish the student with a thorough knowledge of the terminology and also the fundamental principles of the science, which will place him at great advantage in receiving the benefits of clinical instruction.

It is to be regretted that a work, so excellent, should not have been somewhat larger. Several chapters could have been a little more extended without impairing the character of the work as a "handy book."

We most heartily commend this work to the student and the profession at large.

For sale by Keen & Co., 148 Lake st.

---

*Glaucoma and its cure by Iridectomy.* Translated by C. A. ROBERTSON, M. D., of Albany, N. Y. From the French of Testelin & Warlomont.

Glaucoma is undoubtedly a very rare disease in the Northwest. The oculist will scarcely meet with one case in every thousand patients with diseases of the eye he is called upon to examine. A monograph on this affection, therefore, will probably fail to excite interest in the profession. And yet we can assure our readers, that a full discussion of the subject involves so many principles of great value in the study of ophthalmology, that a good monograph on the subject, merits careful study by every physician.

For presenting this translation of such a monograph, Dr. Robertson is deserving of the thanks of the profession.

---

*Orthopædics: A Systematic Treatise vpon the Prevention and Correction of Deformities.* By DAVID PRINCE, M.D.

The very favorable manner in which the medical press generally have received this monograph, is an evidence that its author is very far from failing in his effort to produce an instructive and valuable work.

Dr. Prince is well known as a careful student of his favorite branch of surgery, and as a practitioner, rich in experience.

All who are interested in the subject of orthopœdic surgery will need this work of Dr. Prince.

For sale by Keen & Co., 148 Lake st.

---

*A Practical Treatise on Diseases of the Skin.* By J. MOORE NELIGAN, M. D., etc. Fifth American, from the second revised and enlarged Dublin Edition. By T. W, BELCHER, M. D., Dublin. Philadelphia: Henry C. Lea. 1866.

This instructive little volume appears once more. Since the death of its distinguished author, the study of skin diseases has been considerably advanced, and the results of these investigations have been added by the present editor to the original work of Dr. Neligan. This, however, has not so far increased its bulk as to destroy its reputation as the most convenient manual of diseases of the skin that can be procured by the student.

For sale by W. B. Keen & Co., Chicago.

---

*Cerebro-Spinal Meningitis: Being a Report made to the Illinois State Medical Society, June,* 1866. By J. S. JEWELL, M. D., Professor of Anatomy, Chicago Medical College, etc. etc. Chicago, 1866.

We have examined this treatise with the interest which its worth deserves. Every page gives evidence of thorough study and extensive research. As a well digested abstract of the previous literature of the disease this report has few rivals. The author's deductions and conclusions are marked by rare good sense and discrimination. Hoping that this may not be the Professor's last appearance before the medical public, we can cheerfully recommend his work to all who wish to study the interesting disease which forms its subject.

*An Introduction to Practical Chemistry.* By JOHN E. BOWMAN, F. C. S., late Professor of Practical Chemistry in King's College, London. Edited by CHARLES L. BLOXAM, F. C. S. Fourth American, from the Fifth Revised London Edition. Philadelphia: Henry C. Lea. 1866.

A new edition of this valuable little work.

For sale by W. B. Keen & Co., Chicago.

---

*The Martyrs and Heroes of Illinois in the Great Rebellion.* Biographical Sketches. Edited by JAMES BARNET. Illustrated with Portraits. 2d Edition. Chicago: 1867.

The popularity of this interesting volume is beyond question. It forms an enduring record of the lives of nearly one hundred of our heroes who counted nothing dear in comparison with the love they bore their country. From the great President to the humble private soldier all ranks are represented. Sketches of Surgeons Coatsworth and Lynn of Chicago are also here given, and no one can lay down this memorial without a feeling of tender regret mingling with that pride which in the hearts of his countrymen is the soldier's highest recompense.

For sale by James Barnet, 191 Lake street, Chicago.

---

TICKNOR & FIELDS offer new attractions to the readers of the *Atlantic Monthly.* Prof. O. W. Holmes will furnish another tale for the coming volume, and the best talent of New England will, as heretofore, continue to uphold the reputation of the magazine. The able historical articles which form so prominent a feature of the publication, are alone worth its subscription price. The other serials issued by this house continue to merit the good name which we have always claimed for them.

---

SUBSCRIBERS who desire to have their post-office address changed, must send information to our office at least one month previously. All communications for insertion in the Journal, should be forwarded before the 15th of the month previous to their desired appearance. Letters to which answers are desired must always contain a postage stamp.

